# Prevalence of Alcohol and Tobacco Use among Men and Women in Namibia

**DOI:** 10.3390/ijerph16010059

**Published:** 2018-12-26

**Authors:** Zhifei He, Ghose Bishwajit, Sanni Yaya

**Affiliations:** 1 School of Politics and Public Administration, Southwest University of Political Science & Law, Chongqing 401120, China; houis123@163.com; 2 Faculty of Social Sciences, School of International Development and Global Studies, University of Ottawa, Ottawa, ON K1N6N5, Canada; Sanni.yaya@uottawa.ca

**Keywords:** alcohol, smoking, Demographic and Health Survey, Namibia

## Abstract

Namibia is known to have a high prevalence of tobacco smoking and alcohol consumption. Individuals who smoke are more likely to drink, and vice versa. It was reported that the individual rewarding effect of drinking and smoking were reported to be higher than when they are used at the same time. In this study our objective was to examine the individual and combined prevalence of drinking and smoking and investigate their sociodemographic correlates among adolescent and adult men and women in Namibia. This study was based on data from Namibia Demographic and Health Survey (NDHS 2013). Sample population were 14,185 men and women aged between 15 and 64 years. Self-reported tobacco smoking and alcohol consumption patterns were the outcome variables. Data were analysed using complex sampling techniques to account for survey design. Bivariate and multivariate techniques were used to measure the association between drinking and smoking with the sociodemographic factors. The prevalence of alcohol and tobacco use was, respectively, 53.1% (51.5–54.6) and 8.8% (8.1–9.5), and that of both drinking and smoking was 6.9% (6.3–7.6). In the regression analysis, several sociodemographic factors were found to be significantly associated with alcohol and tobacco use including age, area of residence, religion and educational status. Overall, women had higher rates of drinking alcohol; however, men had higher rates of engaging in high risk drinking. Men and women who reported drinking alcohol had, respectively, 2.57 and 4.60 times higher odds of smoking. Findings suggest that the prevalence of drinking was higher than that of smoking, with men having higher prevalence of high risk drinking. Men and women who drink alcohol were more likely to be smokers. The prevalence of both alcohol and tobacco use showed important sociodemographic patterns which need to be taken into consideration in designing prevention and intervention programs. Strategic tobacco control and smoking cessation approaches should pay particular attention to alcohol users.

## 1. Introduction

Globally, alcohol and nicotine are the most commonly abused drugs and costliest ones in terms of the associated disease burden, healthcare expenses and premature deaths [1,2]. In 2010, tobacco smoking, including passive smoking, and alcohol use were ranked as the leading risk factors for global disease burden by the global Burden of Disease (GBD) study [3]. Approximately 20 per cent of all deaths of adult men and five per cent of deaths of adult women [4] (9% of global) burden is attributed to smoking as tobacco-induced diseases account for an estimated 6.9% of years of life lost and 5.5% of disability adjusted life years [3,5]. Similar to tobacco use, alcohol consumption is also associated with about 200 different diseases, conditions and injuries [6] (as specified in the International Classification of Diseases, Revision 10 [ICD-10]), among which there are about 40 of which alcohol is the sole risk factor [7]. According to the GBD 2010 report, alcohol consumption accounted for about 5% of the global disease burden [8]. The behavioural and sociocultural correlates of tobacco smoking and alcohol consumption were reported for many countries in sub-Saharan Africa; however, not much is known regarding the evidences in Namibian population [9,10,11,12,13]. 

Current evidence suggests that low-and-middle-income countries (LMICs) are experiencing a growing prevalence of alcohol and tobacco use, and are accounting for majority of the tobacco-induced morbidities and mortalities [3,9]. Among the 1.3 billion current smokers worldwide, over 80% are located in the LMICs [4,14]. In the wake of the rising burden of alcohol and tobacco use and associated morbidities, a large volume of epidemiological and policy research has been dedicated to developing preventive measures in the LMICs (e.g., the World Health Organization’s Framework Convention on Tobacco Control or the WHO FCTC). Surtaxing tobacco products and banning tobacco use in public spaces are the examples of some popular tobacco control strategies. Alcohol control strategies by the WHO, on the other hand, have faced more opposing forces within the World Trade Organization (WTO) trade treaties until now. Under the FCTC protocol, the WHO made it a legal requirement for countries to introduce tobacco-control strategies, which is regarded as one of the greatest achievements of the WHO. However, no such efforts were made so far for alcohol policies [15]. 

In recent decades, strong policy commitments and actions in high income countries (HICs) have been able to reduce the prevalence of tobacco use; however, the prevalence in the LMICs are on the rise [10]. One aftermath of this is that the tobacco companies are shifting their production and marketing resources to the LMICs to compensate for the slowing market in HICs [16]. This phenomenon is particularly true for countries in sub-Saharan Africa, where many governments are being lured by foreign investors to adopt tobacco farming as a means to alleviate poverty. In Namibia, for example, billion-dollar tobacco plantation projects are being offered to enhance the economy, while ignoring the negative externalities of tobacco farming for the country’s meagre agricultural resources environment and health of the farming community [17,18]. 

As one of the fastest growing economies, Namibia is undergoing rapid urbanization, demographic transition and changes in lifestyle behaviour, including changing drinking and smoking patterns. In 2010, the country had passed its first Tobacco Products Control Act after decades of strenuous efforts [19], which prohibits smoking in public places including the designated smoking areas, and thus ensuring 100% smoke-free environments in line with the FCTC standards [20]. However, there is no research evidence regarding the efficacy of anti-tobacco programs. To date, there has been no population based survey exploring the sociodemographic factors associated with alcohol and tobacco use in the country [21]. A literature review suggests that the majority of studies concerning alcohol and tobacco use in Africa are based on some of the most prosperous countries e.g., Nigeria [22], Ghana [23], South Africa [24]; however, research on Namibia is remains notably scarce. Lack of quality data on alcohol and tobacco use is a common barrier to evidence-based policy making in Africa. To address this research gap, we conducted this study based on the Namibia Demographic and Health Survey conducted in 2013. The main objectives were to report the prevalence rates across different regions and socioeconomic indicators and to discuss policy options in light of the findings. We hypothesised that there are significant sociodemographic patterns in alcohol and tobacco use, and that alcohol use is associated with higher odds of tobacco use. We also stratified the analysis by sex based on the demonstrated differences in health related behaviour among men and women [11,25].

## 2. Methods

### 2.1. Survey Details

The 2013 Namibia Demographic and Health Survey (NDHS) was the fourth to be conducted in the country as part of the global DHS programme. NDHS serves as a periodic update on a wide range of demographic and health indicators. The survey was implemented by the Ministry of Health and Social Services (MoHSS) in collaboration with the Namibia Statistics Agency (NSA) and the National Institute of Pathology (NIP) with technical assistance by Inner City Fund (ICF International) and financial support from the Government of Namibia, the United States Agency for International Development (USAID) and the Global Fund. For sampling, a two-stage stratified technique was employed. In the first stage, 554 enumeration areas (primary sampling units) were randomly selected from urban (269) and rural areas (285). In the second stage, a fixed number of households (20) were selected from each of the urban and rural clusters. From the selected households, a total of 10,861 women and 5271 men were identified as eligible for interview, of whom the final interview was completed by 10,018 women (response rate 92%) and 4481 men (response rate 85%). Field work for the survey lasted from May to September of 2013. More information regarding the survey is available in the DHS report [26]. 

### 2.2. Variables

The outcome variable was self-reported alcohol and tobacco consumption. Lifetime tobacco (Ever smoked vs. Never smoked) and alcohol use (Ever drank alcohol vs. Never drank alcohol) history was collected during the interview for both male and female participants. A small proportion of male participants did not provide information on drinking and smoking behaviour (*n* = 314), and were excluded from the analysis (total final sample = 14,185).

We additionally calculated the prevalence of high-risk drinking among men and women in urban and rural areas. High-risk drinking was defined as >14 drinks per week for men and >7 for women, according to the guidelines by The National Institute on Alcohol Abuse and Alcoholism (NIAAA) [27]. Given the small proportion of the sample providing information on drinking frequency, only descriptive analyses were conducted with these outcomes.

### 2.3. Covariates 

The following variables were included in the analysis based on their demonstrated association with drinking and smoking behavior: Age groups (15–19/20–24/25–29/30–34/35–39/40–44/45+); Residency (Urban/Rural); Sex (Male/Female); Marital status (Never in union/Married/in union/Divorced/other); Education (No education/Primary/Secondary/Higher); Religion (Christian/Elcin/other); Employment (Unemployed/Professional-technical-managerial Agriculture/other); Wealth status * (Poor-Non-poor) [22,24,28,29,30]. We also measured the prevalence of alcohol and tobacco use by Region (Caprivi, Erongo, Hardap, Karas, Khomas, Kunene, Ohangwena, Omaheke, Omusati, Oshana, Oshikoto, Otjozondjupa).

*Wealth status was measured in all DHS surveys based on household ownership of durable goods, which were taken into account for calculating wealth scores by principle component analysis. The scores were then categorised into quintiles, with higher quintiles representing higher wealth status. For this study, wealth quintiles were merged into two categories: Q1 + Q2 = Poor, Q3 + Q4 + Q5 = Non-poor.

### 2.4. Data Analysis

Data were analysed with SPSS 24. Before analysis, the datasets merged (for men and women) and cleaned for missing values and outliers (values beyond the expected measures). In order to account for the cluster sampling design, sampling strata and weight were applied by using a complex survey mode. The prevalence of drinking and smoking along with the control variables were described by percentages with 95% Confidence Intervals. Regional differences in alcohol and tobacco consumption were calculated by cross-tabulation and were presented as bar charts. Finally, a series of logistic regressions were performed to calculate the odds ratios of the associations between alcohol and tobacco, as well as the use of both with the covariates. All tests were two-tailed and were considered significant at alpha value of 5%. 

Ethical clearance: ethical approval was not necessary for this study as the data were secondary and are available in public domain in anonymised form.

## 3. Results

The sample population comprised of 4167 men and 10,018 women. Mean age of the men was 31.89 years (95% CI = 31.52–32.27) and that of women was 31.36 years (31.13–31.60). Findings suggested that the prevalence of drinking was far higher than that of smoking. The overall prevalence of drinking and smoking was, respectively, 8.8% (8.1–9.5) and 53.1% (51.5–54.6), and that of both drinking and smoking was 6.9% (6.3–7.6). Prevalence of smoking was 64.09% and 35.91%, and that of alcohol consumption was 35.19% and 64.81%, respectively, among men and women (not shown in the analysis). The basic sociodemographic profiles of the participants according to drinking and smoking habits is summarised in Table 1. The prevalence of both tended to be higher among those aged 20–24, residence of urban areas, among male respondents (except for alcohol consumption being higher among women), who had secondary/higher education, who were followers of Christianity (except for alcohol consumption), had no employment and lived on non-poor households.

**Figure 1:**
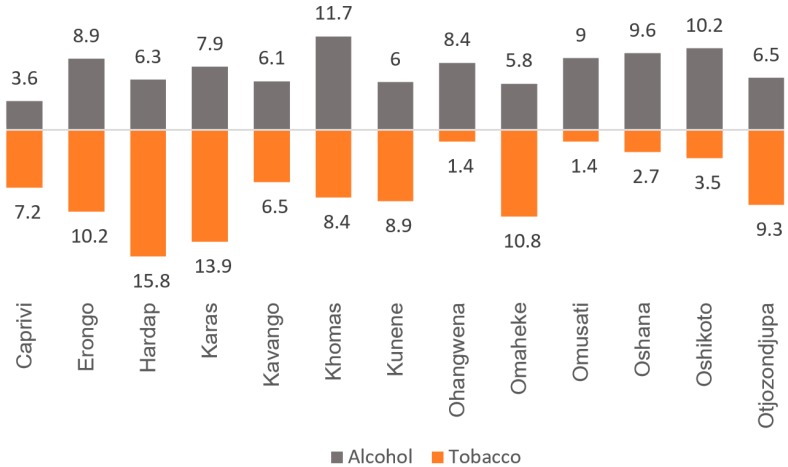
Regional prevalence of lifetime alcohol consumption and tobacco smoking in Namibia.

Figure 1 depicts the regional disparities in alcohol and tobacco use in Namibia. The prevalence of smoking ranged from as high as 15.8% in Hardap to 1.4% in Ohangwena and Omusati, whereas that of alcohol consumption ranged from 11.7% in Khomas to 3.6% in Caprivi. In general, regions with lower rates of alcohol use also had a relatively lower rate of tobacco use.

**Figure 2:**
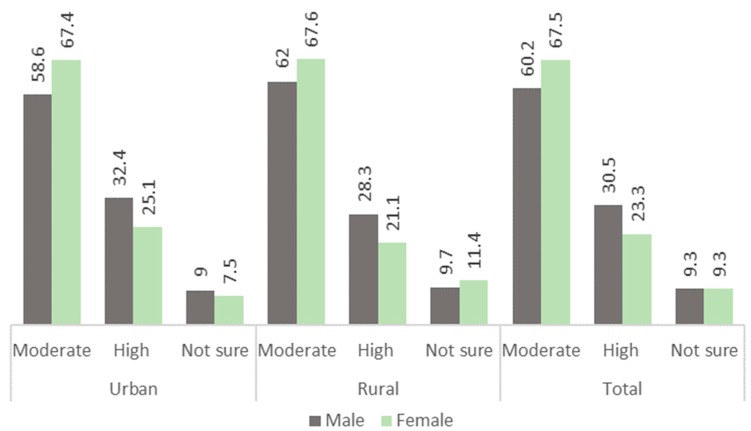
Prevalence of moderate- and high-risk drinking in past 2 weeks (*n* = 3779).

Figure 2 shows the prevalence of moderate high-risk drinking (>7 drinks/week for women and >14 drinks/week for men) among men and women during the last weeks of the survey. Overall, women had higher rates of drinking alcohol; however, men had higher rates of engaging in high risk drinking. 

## 4. Regression Analysis on the Association between Alcohol and Tobacco Use with Sociodemographic Factors

As shown in Table 2, adolescent boys and girls (aged 15–19 year) had significantly lower odds of both drinking and smoking compared with men and women in the highest age category, whereas participants in the age groups in between had higher odds of drinking, except for those aged 40–44 years. Overall, men had high odds of smoking, drinking and both than women. Men in urban areas were more likely to be smokers than women; on the other hand, urban women were more likely to ever drink than men.

Additionally, we checked whether the odds of smoking were significantly associated with that of drinking. The results in Table 3 indicate that both men and women who reported drinking alcohol had significantly higher odds of smoking tobacco.

## 5. Discussion and Policy Recommendation

In this study, we found that a great majority of Namibian adults reported about consuming alcohol at any point of their life, and a significant proportion with high-risk level of drinking in the past two weeks. More than half of participants reported ever consuming alcohol, in contrast less than one-tenth reported ever smoking. There was no information on current habit or pattern of smoking, although a small proportion of the sample reported it on their recent drinking activities. The finding that the prevalence of ever consuming alcohol was far lower than that of smoking might be explained by the continued policy effort to ban smoking in public places under the FCTC framework. The survey data used in this study were collected about three years following the enactment of the anti-tobacco policy, and thus, do not necessarily reflect the current situation. No data were available before the anti-tobacco policy came into force. Therefore, it is hard to deduce whether the prevalence of smoking has decreased since. Nonetheless, the remarkably lower prevalence of smoking than alcohol consumption can be assumed to be an important lesson. Currently, there is no quality evidence on alcohol consumption behaviour and alcohol-attributable diseases burden in Namibia. More studies are necessary to investigate dinking patterns and explore policy options for anti-alcohol campaigns and their efficacy on reducing the control the level of consumption.

The findings of this study can have important messages for tobacco and alcohol control strategies in Namibia as well as in the neighbouring countries. Significant variation was observed for drinking and smoking across the sociodemographic categories. In line with the first hypothesis, the results revealed a significant sociodemographic pattern in both alcohol and tobacco use. As expected, the prevalence of both alcohol and tobacco use was highest among the young age groups (20–29 years). The prevalence increased from adolescent groups until the age group of 30–34 years, and then decreased to be lowest among the eldest groups. Urban men and women had significantly higher prevalence of alcohol and tobacco use compared with rural residents. This is possibly due to better socioeconomic status, exposure to electronic media and advertisements. The fact that education showed no protective effect on alcohol and tobacco might be indicative of low public awareness and/or active efforts to abstain from these habits. Current literature suggests that the relationship between education and smoking is not a linear one and is impacted by a wide range of psychosocial factors [28,31,32]. Previous studies have also reported the educational gradient in smoking is increasing, with a widening gap in smoking rates between individuals with the highest and lowest levels of educational qualification [28,33]. Findings showed that participants from non-poor households and comparatively higher educational status had higher prevalence of drinking and smoking. These findings regarding to sociodemographic and economic disparities in tobacco use are similar to those reported in Ethiopia and Malawi [32] and Ethiopia [34]. Regarding the association between alcohol and tobacco use, the findings also supported the second hypothesis that alcohol use is associated with significantly higher odds of using tobacco. The evidence on the concurrent effect of drinking and smoking and other drug abuse has been demonstrated in previous studies in developed countries [35,36]. Our findings support the past evidence and report that the association is true in low-income settings as well.

The apparently surprising positive association between education and ever drinking alcohol was reported in earlier studies from high-income settings [37,38,39]. However, the evidence cannot help reach any concrete conclusion as the studies vary greatly in terms of methodological approaches (e.g., type/strength of beverages consumed). It is possible that people with higher socioeconomic status are less likely to have addictive behaviour and more likely to involve in social drinking with higher frequency but with lower volume of drinking [39]. Individual drinking and smoking behaviour varies substantially according to sociocultural contexts. For instance, drinking has been considered a more salubrious and socially acceptable behaviour in some cultures, which makes policy approaches particularly challenging [15]. Regardless of environmental influences, educational interventions for drinking and smoking behaviour have proven to be effective in many countries. Policy interventions for alcoholic beverages are less common compared with tobacco industry, which might explain for the rising rate of alcohol use in Africa.

Lack of country-specific data makes it hard to understand the root causes of alcohol and tobacco use behaviours. However, existing surveys indicate that the increasing influence of tobacco companies in the LMICs might be triggering the rising trend in smoking. Compared to HICs, exposure to tobacco advertisements were reported to be 81 times higher in LMICs, where people were 46 times more likely to encounter tobacco adverts in radio, 11 times more likely from posters and nine times more for television [40]. At the same time, there has been a considerable reduction in tobacco use in high income countries [7,8]. To compensate the declining market, the tobacco industry is gradually shifting their market and resources to the LMICs where about 80% of the world’s smokers reside [29]. Governments in poor countries in sub-Saharan Africa often tend to be lenient to tobacco investors, such as in Namibia and Zimbabwe. Stronger efforts by health experts and politicians will be necessary to make sure that the national policies are in line with the public health goal of the country. 

This study is the first to report the prevalence and sociodemographic factors of alcohol and tobacco use in Namibia. The data were secondary, and hence, cannot represent the current prevalence rates; nonetheless, the findings carry several important messages that can prove to be useful for alcohol and tobacco prevention programs in Namibia. Apart from the findings, there are some important strengths and limitations to note. Firstly, the data were of high quality and representative of the population. Both men and women were included in the study within a substantially wide age range, which helped to understand the sex and age differentials in the prevalence rates. However, the data being secondary also meant that we had no control over the selection and measurement of the variables. For example, we could not analyse the cultural and behavioural factors of smoking. Additionally, as it was a cross-sectional survey, the findings cannot help make any causal inference. The information on drinking and smoking were collected subjectively, and therefore, remain subject to recall bias. 

## 6. Conclusions

In conclusion, the findings of the present study revealed that a great majority of the men and women are used to alcohol consumption, compared to one-tenth for tobacco smoking. Additionally, about a quarter of the population might be exposed to heavy drinking behaviour. Several sociodemographic factors were found to be significantly associated with drinking and smoking such as age, type of residency, religious affiliation and educational attainment. More studies are required to explore the sociocultural and policy-level factors that may be contributing to the high rates of alcohol users.

## Figures and Tables

**Table 1:** Basic profile of the participants. (*n* = 14,185).

		Smokes Tobacco		Drinks Alcohol	
	N, %	Yes 8.8% (8.1–9.5)	No 91.2% (90.5–91.9)	Yes 53.1% (51.5–54.6)	No 46.9% (45.4–48.5)
**Age groups**					
15–19	2740, 19.3	6.7 (5.1–8.6)	22.5 (21.5–23.6)	15.4 (14.2–16.7)	27.6 (26.1–29.2)
20–24	2491, 17.6	19.0 (16.5–21.6)	19.4 (18.6–20.3)	21.0 (19.7–22.3)	17.6 (16.4–18.8)
25–29	2108, 14.9	18.9 (16.7–21.3)	15.8 (15.0–16.6)	17.9 (16.9–18.9)	14.0 (12.9–15.0)
30–34	1778, 12.5	15.5 (13.1–18.3)	13.1 (12.4–13.9)	14.5 (13.5–15.5)	12.0 (11.1–13.0)
35–39	1600, 11.3	14.3 (12.3–16.7)	11.4 (10.7–12.1)	12.7 (11.8–13.7)	10.5 (9.5–11.5)
40–44	1346, 9.5	13.5 (11.4–15.9)	9.3 (8.7–9.9)	9.6 (8.8–10.5)	9.7 (8.9–10.6)
45+	2122, 15	12.2 (10.3–14.4)	8.5 (7.9–9.2)	9.0 (8.3–9.8)	8.6 (7.8–9.6)
*p*-value		<0.001		<0.001	
**Residency**					
Urban	7256, 51.2	65.3 (61.6–68.9)	55.7 (53.9–57.5)	57.7 (55.5–59.8)	55.2 (52.9–57.5)
Rural	6929, 48.8	34.7 (31.1–38.4)	44.3 (42.5–46.1)	42.3 (40.2–44.5)	44.8 (42.5–47.1)
*p*-value		<0.001		<0.001	
**Sex**					
Male	4167, 29.4	67.3 (64.0–70.5)	28.0 (26.8–29.2)	36.0 (34.3–37.7)	26.3 (24.8–27.8)
Female	10018, 70.6	32.7 (29.5–36.0)	72.0 (70.8–73.2)	64.0 (62.3–65.7)	73.7 (72.2–75.2)
*p*-value		<0.001		<0.001	
**Marital status**					
Never in union	6054, 42.7	48.2 (45.0–51.5)	43.7 (42.3–45.1)	45.2 (43.4–47.1)	42.8 (41.3–44.4)
Married/in union	6970, 49.1	45.2 (42.1–48.3)	48.0 (46.7–49.4)	47.0 (45.2–48.8)	48.7 (47.1–50.3)
Divorced/other	1161, 8.2	7.8 (7.0–8.6)	8.4 (7.6–9.3)	6.6 (5.2–8.2)	8.3 (7.6–8.9)
*p*-value		0.323		0.271	
**Education**					
No education	1142, 8.1	10.0 (8.3–12.0)	5.3 (4.6–6.0)	5.4 (4.6–6.3)	6.0 (5.3–6.8)
Primary	3356, 23.7	23.6 (20.8–26.6)	20.7 (19.5–22.0)	19.8 (18.3–21.4)	22.3 (20.8–23.8)
Secondary/Higher	9687, 68.3	66.4 (64.6–69.4)	74 (72.3–78.3)	74.8 (70.7–78.7)	71.7 (69.7–76.1)
*p*-value		<0.001		<0.001	
**Religion**					
Christian	5923, 41.8	44.2 (40.5–48.0)	39.7 (37.9–41.6)	40.2 (38.1–42.3)	40.0 (38.0–42.0)
Elcin	5793, 40.8	31.7 (28.2–35.5)	44.9 (42.9–47.0)	47.1 (44.8–49.4)	40.0 (37.8–42.3)
Other	2469, 17.4	24.1 (20.8–27.7)	15.4 (14.0–16.9)	12.7 (11.3–14.2)	20.0 (18.2–21.9)
*p*-value		<0.001		<0.001	
**Employment**					
Unemployed	7142, 50.3	51.1 (49.5–52.7)	31.8 (28.5–35.4)	42.5 (40.6–44.4)	57.2 (55.6–58.9)
Professional/ technical /managerial	3746, 26.4	27.2 (25.9–28.4)	29.8 (26.4–33.4)	30.8 (29.0–32.6)	23.6 (22.3–24.9)
Agriculture/ other	3297, 23.2	21.7 (20.4–23.1)	38.4 (34.8–42.1)	26.7 (25.0–28.6)	19.2 (17.9–20.5)
*p*-value		<0.001		<0.001	
**Wealth status**					
Poor	4896, 34.5	33.7 (31.2–36.2)	30.3 (26.4–34.5)	31.6 (28.8–34.5)	35.4 (32.8–38.2)
Non-poor	9289, 65.5	66.3 (63.8–68.8)	69.7 (65.5–73.6)	68.4 (65.5–71.2)	64.6 (61.8–67.2)
*p*-value		0.306		0.04	

**Table 2:** Odds ratios of association between alcohol and tobacco use with sociodemographic variables in Namibia. (Namibia Demographic and Health Survey 2013).

	Alcohol Consumption			Tobacco Smoking			Alcohol & Tobacco		
	Total	Men	Women	Total	Men	Women	Total	Men	Women
**Age groups**									
15–19	0.660 *	0.605 *	0.686 *	0.242 *	0.227 *	0.272 *	0.262 *	0.212 *	0.365 *
20–24	1.259 *	1.414 *	1.175	0.85	0.917	0.703	0.946	1.023	0.771
25–29	1.309 *	1.532 *	1.225	0.988	1.095	0.816	1.070	1.171	0.898
30–34	1.212 *	1.318	1.178	0.999	1.141	0.802	1.031	1.076	0.965
35–39	1.221 *	1.186	1.232	1.092	1.127	1.039	1.181	1.24	1.156
40–44	0.989	0.892	0.965	1.248	1.095	1.431	1.180	1.010	1.435
45+	Ref	Ref	Ref	Ref	Ref	Ref	Ref	Ref	Ref
**Residency**									
Urban	0.902	1.269 *	1.036	1.463 *	1.093	2.460 *	1.549 *	1.161	2.603 *
Rural	Ref	Ref	Ref	Ref	Ref	Ref	Ref	Ref	Ref
**Sex**									
Male	0.801 *	-	-	5.447 *	-	-	4.825 *	-	-
Female	Ref	Ref	Ref	Ref	Ref	Ref	Ref	Ref	Ref
**Education**									
Secondary	1.854 *	1.754 *	0.576 *	3.221 *	2.184 *	5.873 *	2.918 *	1.951 *	5.519 *
Higher	1.599 *	1.717	0.563 *	2.872 *	2.443 *	3.352 *	2.487 *	2.022 *	3.148 *
None	Ref	Ref	Ref	Ref	Ref	Ref	Ref	Ref	Ref
**Religion**									
Christian	1.692 *	1.656 *	1.743 *	0.750 *	0.648 *	1.009	1.776 *	1.698 *	0.972
Elcin	1.949 *	1.839 *	2.047 *	0.442 *	0.41 *	0.561 *	0.477 *	0.468 *	0.546 *
Other	Ref	Ref	Ref	Ref	Ref	Ref	Ref	Ref	Ref
**Employment**									
Agriculture/other	0.723 *	0.565 *	0.844 *	0.876	0.739 *	1.326	0.754 *	0.625 *	1.141
Professional/technical/managerial	1.039	1.117	1.082	1.140	0.965	1.476 *	1.148	1.040	1.445
Unemployed	Ref	Ref	Ref	Ref	Ref	Ref	Ref	Ref	Ref
**Wealth status**									
Poor	0.934	0.985	0.905	0.898	0.957	0.799	0.934	1.021	0.785
Non-poor	Ref	Ref	Ref	Ref	Ref	Ref	Ref	Ref	Ref

N.B: * = significant at *p* < 0.05.

**Table 3:** Odds ratios of smoking among men and women according to drinking status.

	Total	Men	Women
Drink alcohol			
No	Ref	Ref	Ref
Yes	3.565 *	2.573 *	4.603 *

N.B: * = significant at *p* < 0.05.
